# Harnessing the stem cell potential in the human hippocampus to limit cognitive aging

**DOI:** 10.1371/journal.pbio.3003787

**Published:** 2026-05-22

**Authors:** Susmit Mhatre, Darcie L. Moore

**Affiliations:** 1 Department of Neuroscience, University of Wisconsin-Madison, Madison, Wisconsin, United States of America; 2 Cell and Molecular Biology Graduate Program, University of Wisconsin-Madison, Madison, Wisconsin, United States of America; Harvard University T H Chan School of Public Health, UNITED STATES OF AMERICA

## Abstract

The field of human adult neurogenesis has been controversial despite mounting evidence. This Perspective proposes moving beyond debating the existence of adult neurogenesis, and towards discovering strategies to harness endogenous stem cell potential for resilience against cognitive aging.

## Teaser

The field of human adult neurogenesis has been controversial despite mounting evidence. The authors propose moving beyond debating the existence of adult neurogenesis and towards discovering strategies to harness endogenous stem cell potential for resilience against cognitive aging.

Cognitive decline is an unwelcome part of "normal" aging. Memory fades gradually: forgetting why we went to the refrigerator or leaving the dog outside, calling family members by other names. We begin to ask ourselves: are these the first steps towards losing the memories of a well-lived life—and what, if anything, can we do?

In rodents, new neurons can still be produced in adults in the brain region important for learning and memory, the hippocampus. These new neurons arise from a population of dormant stem cells. When signaled, these cells divide and make daughter cells that ultimately produce these newborn neurons that integrate into the existing circuitry, a process called adult neurogenesis. Though the numbers of stem cells are low, and the newborn neurons produced are few, the increase or loss of these cells is tightly related to specific cognitive functions. In the aged brain, fewer newborn neurons are produced, and increasing adult neurogenesis in the context of aging improves memory and cognition in these animals. Could this be a target for improving cognitive function in aging humans?

One might think that pursuing this research in humans makes perfect sense. However, for reasons that are still unclear, the field of adult neurogenesis has been controversial since Ramón y Cajal’s establishment of the dogma of “no new neurons” almost a century ago [1]. Even in rodents, the concept of adult neurogenesis was initially disputed. Now, after 60 years of the development and use of more advanced technologies expanding our understanding [2], there are none who disagrees that adult neurogenesis in the rodent brain exists. However, when studies began in non-human primate hippocampus, there was once again controversy, which finally resulted in agreement that adult neurogenesis occurred there, albeit at lower levels than in rodents [3]. Yet the debate still did not end. It simply shifted from whether it occurs in any mammals to whether it occurs in humans. Somewhere along the way, the disagreement became the focus, rather than the biology.

In 2019, the Society for Neuroscience hosted a Dual Perspectives session on human adult hippocampal neurogenesis in a room that was so packed it likely violated fire and building codes. Yet at its core, this debate focused on the technical differences between immunostaining protocols. Indeed, technical details seem to be where the greatest controversy lies. In fact, one can summarize the scientific arguments for adult neurogenesis in humans by making generalized groupings of contributing studies based on technical approaches (Table 1). All of these approaches are similarly hindered by the comparatively small population of neural stem cells and newborn neurons in the adult brain, their tight compaction in a highly populated mature dentate gyrus, and their similarities to astrocytes, or mature neurons, respectively. With the underlying arguments being centered on so much technical detail, how can those outside of the field, including patients and clinicians with a genuine stake in the answer, navigate these conflicting reports?

**Table 1:** Arguments for and against human adult hippocampal neurogenesis across experimental approaches.

Technique	Argument against adult hippocampal neurogenesis	Argument for adult hippocampal neurogenesis
**Immunostaining**	New neurons were found in the hippocampus of children but not adults with similar postmortem tissue processing. DCX^+^ cells are found in other brain regions and may not be a marker of newborn neurons.	Detection of sensitive antigens such as DCX and PSA-NCAM varies substantially with tissue quality and history. With appropriate antigen retrieval and fixation conditions, consideration for age, post-mortem interval, psychological status, neural stem cells, neuroblasts, and newborn neurons were detected in adult hippocampal brain sections.
^**14**^ **C birthdating**	^14^C incorporation in NeuN^+^ nuclei in the hippocampus could potentially indicate post-mitotic neurons re-entering the cell cycle or undergoing DNA repair or methylation. Subpopulations of oligodendrocytes and microglia, and not just neurons, can also be isolated as NeuN^+^ nuclei.	High ^14^C levels in genomic DNA of NeuN^+^ nuclei could only be explained by newborn neurons generated throughout adulthood in humans. ^14^C integration by DNA damage and repair is several magnitudes below mitotic cells. Neurons of non-neurogenic regions such as cortex, cerebellum, or olfactory bulb do not show elevated ^14^C levels.
**Transcriptomics**	Cross-species examination with combinatorial gene expression using single-nuclear RNA sequencing could detect trajectories for adult neurogenesis in mouse, pig, and macaque, but not in humans.	Rodents, pigs, and macaques have divergence in gene expression from humans. Using machine learning-based algorithms and fetal and postnatal human samples, single-nuclear RNA sequencing identifies neural stem cells, progenitors, and immature granular cells in hippocampus, with confirmation at the level of spatial transcriptomics.
***In vitro* cultures**	Proliferation *in vitro* under nutrient-rich growth factor-supplemented conditions do not accurately mirror the prevalent state of quiescence of supposed neural stem progenitor cells in adult human hippocampus *in vivo*. Proliferating cells *in vitro* may come from a different cell type.	Harvested cells from human post-mortem hippocampus, and not cortex, can be cultured *in vitro* revealing stem cell behaviors: self-renewal through neurosphere formation, and differentiation into neurons and glial cells.

The evidence, when viewed together, is more coherent than the debate suggests. In our view, the most rigorous, well-controlled immunostaining papers (see [4] for a meticulous supplementary figures section) have effectively shown newborn neurons in the human hippocampus. RNA sequencing approaches using human fetal molecular signatures of newborn neurons and stem cells, rather than relying on rodent-based signatures, revealed these rare populations in the adult [5,6]. When samples from individuals who were young, aged, a ‘SuperAger’ (an individual above 80 years old with a memory performance of 2–3 decades younger), or had Alzheimer’s disease (AD) were compared, neural stem cells and newborn neurons changed in the direction expected [7]. ^14^C studies detected neuronal turnover in the hippocampus, but not in the cortex [8]. And using resected hippocampus surgically removed from epilepsy patients, cells have been cultured that produce stem cell-containing neurospheres that can be differentiated into neurons, astrocytes, and oligodendrocytes, demonstrating self-renewal and multipotency, respectively—the defining properties of a neural stem cell [9]. Whether this stemness arrived from a traditional neural stem cell population or a different cell type is not clear, but the fact that this tissue has stem cells and can make neurons *in vitro* effectively answers the question of whether there is stem cell potential in the human hippocampus. Despite the continued divergent opinions on the topic, the science has moved on, even if the argument has not.

Skeptics of whether adult neurogenesis occurs often focus on technical concerns that are different for each approach [10]. Or ask why, if neurogenesis is abundant, aged hippocampi are not bigger, or why neuronal death is not rampant? How can so few neural stem cells and newborn neurons have any effect on behaviors? All of these are reasonable questions and require additional experimentation to understand further, particularly as technical advances continue, but these questions do not mean that human adult neurogenesis does not exist, nor that resources and effort should be limited to study it.

What is adult neurogenesis good for anyway? In rodents, where experiments can be well controlled with targeted ablation, these newborn neurons contribute to new learning, reduced anxiety, pattern separation, spatial learning, updating stored information, generalization, and improved cognitive flexibility [11]. In humans, perhaps the role of newborn neurons may not be as pronounced in a young brain, but in the context of age or disease, what could it mean for these functions if we are able to increase neurogenesis even at low levels? A recent study using RNA sequencing in humans reported that SuperAgers have a higher number of newborn neurons compared to aged adults, and patients with AD had a decreased number of newborn neurons, suggesting that the levels of neurogenesis correlate with cognitive function in aged conditions [7]. Of course, due to inherent limitations with human studies, we cannot establish a causative relationship between cognition and neurogenesis, at least not yet. Additionally, most researchers acknowledge that adult neurogenesis levels may not be the whole story, especially in disease. There may be synergies that are required in these conditions that we have not yet explored. For example, in a rodent model of AD, increasing both adult neurogenesis and levels of the growth factor BDNF improved cognition in these animals, but increasing adult neurogenesis alone in the diseased environment was not enough to improve cognitive deficits [12]. This tells us it is more complex than we understand right now, which means we need more research into this topic, not less.

But even without those human-specific answers, what we know from rodents suggests that increasing neurogenesis may improve brain health in humans and promote healthy aging. Aged rodents running on wheels, an intervention known to potently increase neurogenesis, showed improved memory performance [13]. Those housed in enriched environments showed both more newborn neurons and improved recall [14]. Together, these findings point toward a converging set of lifestyle factors worth considering. Physical exercise may be the most straightforward place to start. Novelty matters too: regular exposure to new environments may engage and support hippocampal function in ways that accumulate over time. Cognitive engagement is similarly promising—activities that draw on learning, memorization, and pattern recognition can increase the survival and integration of newborn neurons in the hippocampus of rodents. Finally, chronic stress is one of the strongest negative regulators of neurogenesis and is associated with anxiety, social avoidance, and mood dysregulation [15]. Managing stress might be as important for cognitive aging as any positive intervention. While a thorough understanding of these interventions in humans remains limited, the convergence across exercise, novelty-seeking, cognitive engagement, and stress regulation is striking—and within our reach.

Where do we go from here? We can begin by incorporating these interventions into our lives now, knowing they benefit many aspects of our health well beyond cognition. Research must continue to determine how we can harness the regenerative capacity in the aging brain. We cannot harness what we refuse to acknowledge exists, so let us work together to resolve the technical challenges of studying adult neurogenesis in humans and discover new ways to preserve our cognitive abilities with age. The memories of a well-lived life, and the independence to keep living it, are worth every effort to understand and preserve.

## Abbreviation

AD: Alzheimer’s disease
